# Performance of fractional exhaled nitric oxide in predicting response to inhaled corticosteroids in chronic cough: a meta-analysis

**DOI:** 10.1080/07853890.2021.1979242

**Published:** 2021-09-16

**Authors:** Pasquale Ambrosino, Mariasofia Accardo, Marco Mosella, Antimo Papa, Salvatore Fuschillo, Giorgio Alfredo Spedicato, Andrea Motta, Mauro Maniscalco

**Affiliations:** a Istituti Clinici Scientifici Maugeri IRCCS, Pavia, Italy; b Department of Data Analytics and Actuarial Science, Unipol Group, Bologna, Italy; c Institute of Biomolecular Chemistry, National Research Council, ICB-CNR, Naples, Italy

**Keywords:** Fractional exhaled nitric oxide, chronic cough, chronic disease, disability, rehabilitation, exercise, outcome

## Abstract

**Background.** Chronic cough is a disabling condition with a high proportion of diagnostic and therapeutic failures. Fractional exhaled nitric oxide (FeNO) has been considered a useful biomarker for predicting inhaled corticosteroids (ICS) response. We evaluated the relationship between FeNO and ICS response in chronic cough by performing a systematic review with meta-analysis.

**Methods.** PubMed, Web of Science, Scopus and EMBASE databases were systematically searched. Differences were expressed as Odds Ratio (OR) with 95% confidence intervals (95%CI). Pooled sensitivity, specificity, positive (PLR) and negative likelihood ratio (NLR), and area under the hierarchical summary receiver operating characteristic curve (HSROC_AUC_) were estimated.

**Results.** Nine articles on 740 chronic-cough patients showed that the response rate to ICS was 87.4% (95%CI: 83.8–91.0) in 317 patients with a high FeNO and 46.3% (95%CI: 41.6–51.0) in 423 controls, with an attributable proportion of 47.0% and a diagnostic OR of 9.1 (95%CI: 3.7–22.4, *p* < .001). The pooled estimate of diagnostic indexes resulted in a sensitivity of 68.5% (95%CI: 46.7–84.4) and specificity of 81.9% (95%CI: 63.0–92.3), with a HSROC_AUC_ of 0.82 (95%CI: 0.64–0.90). In a realistic scenario with a pre-test probability set at 30%, based on a pooled PLR of 3.79 (95%CI: 1.24–7.47) and NLR of 0.38 (95%CI: 0.22–0.66), the post-test probability was 62% with a high FeNO and 14% if the test was negative. Subgroup analyses confirmed a better performance for the recommended FeNO cut-off greater than 25 ppb. Meta-regression and sensitivity analyses showed no impact of major demographic and clinic variables on results.

**Conclusions.** A high FeNO before starting ICS therapy may help identify chronic-cough patients responding to treatment, with a better performance ofhigher cut-off values. Further studies are needed to evaluate the real usefulness of this biomarker to guide cough therapy and optimise strategies in different healthcare settings (community, hospital, rehabilitation).

**Key messages**
- Chronic cough is a disabling condition with a high proportion of diagnostic and therapeutic failures.
- Fractional exhaled nitric oxide (FeNO) may be a useful biomarker for identifying chronic cough patients who respond to steroid treatment.
- A FeNO cut-off lower than 25 ppb should be considered irrelevant for this clinical application.

## Introduction

With a worldwide prevalence of 9.6% [1], chronic cough is defined as a cough that lasts for more than 8 weeks [2], resulting in substantial disability and quality of life impairment [3]. Several diseases may be responsible for this clinical manifestation, including asthma, eosinophilic bronchitis, pulmonary fibrosis, gastro-esophageal reflux disease (GERD), postnasal drip syndrome (PNDS), chronic obstructive pulmonary disease (COPD), and respiratory tract infections (RTI) [4]. Exposure to cigarette smoke, environmental pollution and blood pressure medications are also involved in chronic cough pathogenesis [5].

Up to 44% of diagnostic and therapeutic failures have been documented among patients with chronic cough [3,6], thus leading to the new paradigm of a neuropathic syndrome with its own pathophysiology, known as cough hypersensitivity syndrome (CHS) [7]. In parallel, such diagnostic and therapeutic challenges called for the development of specific guidelines for chronic cough aimed at improving the current management strategies [4–6]. Such guidelines all rely on a common principle based on the identification and treatment of the disease potentially responsible for this clinical manifestation.

Among the peripheral stimuli for cough reflex, eosinophilic airway inflammation is one of the most frequent and, consequently, one of the most studied in terms of pathophysiology and clinical implications [7]. Having this airway inflammation a good response to inhaled corticosteroids (ICS) [8], it is crucial to identify potential ICS responders to enable tailored therapeutic strategies and avoid treatment failures or adverse drug reactions [2]. Conventionally, induced sputum eosinophil count has been used to diagnose eosinophilic airway inflammation, thus driving decisions on ICS treatment in patients with asthma, eosinophilic bronchitis and atopic cough [9]. However, this method is technically and logistically challenging, being currently restricted to a limited number of specialised centres.

Past observation that the nitric oxide (NO) concentration in exhaled air generally increases in chronic inflammatory airway diseases [10,11] has led the European Respiratory Society (ERS) and the American Thoracic Society (ATS) to agree on highly standardised procedures to measure fractional exhaled NO (FeNO) [12–14]. Thus, FeNO has become an important non-invasive support to monitor compliance and efficacy of steroids in chronic airway diseases, especially asthma [15,16]. More recently, the potential use of FeNO in predicting ICS response in chronic cough patients has attracted an increasing interest. In particular, some studies documented a higher response rate to ICS among chronic cough patients with a high FeNO as compared to those with a low FeNO [17,18], thus suggesting its potential role in guiding decisions on ICS initiation. However, recent data have challenged these results [19,20], and no meta-analytical data providing a comprehensive information about this issue are currently available.

Here, we performed a systematic review with meta-analysis and meta-regressions to evaluate the association between high FeNO and ICS response in chronic cough.

## Methods

For this systematic review, we prospectively developed a protocol, specifying the objectives, the principles for the studies’ selection, the method for study quality assessment, the outcomes and statistical methods. The review protocol was registered on PROSPERO with identifier CRD42021261847.

### Search strategy

In agreement with the Preferred Reporting Items for Systematic Reviews and Meta-Analyses (PRISMA) guidelines [21], we performed a systematic search in electronic databases (PubMed, Web of Science, Scopus, EMBASE). The search terms *steroid, steroids, corticosteroid, corticosteroids, glucocorticoid, glucocorticoids, cough,* and *nitric oxide* were used in all possible combinations, without language restriction. The last search was performed on June 8, 2021.

In addition, the lists of the retrieved articles were manually reviewed. For missing data, we tried to contact the authors to obtain the original data. Two investigators (MMo and MA) independently analysed the studies and carried out data extraction. For divergent opinions, a third investigator was consulted (SF). Discrepancies were resolved by consensus. Selection results presented a high inter-reader agreement (κ = 0.97), and have been detailed according to PRISMA flowchart (Supplemental Figure 1).

### Data extraction and quality assessment

According to the established protocol, we considered all studies reporting the rate of ICS response in chronic cough subjects with high and low FeNO. Case-reports, case-series without a control group, reviews and animal studies were not included. We also evaluated abstracts and citations from scientific conferences. The data extracted from the studies were study design, sample size, major clinical and demographic variables, number of subjects with a high FeNO (cases), number of subjects with a low FeNO (controls), and rate of ICS response in each of the two groups. Data on FeNO values (mean with standard deviation or standard error) among responders and non-responders were also collected. Devices and methods for FeNO assessment and FeNO cut-off values used in included studies are reported in Supplemental Table 1.

Quality Assessment of Diagnostic Accuracy Studies 2 (QUADAS-2) was used to evaluate the methodological quality of each study. This tool was specifically designed to judge the quality of diagnostic accuracy studies [22]. In brief, the scoring system considers 2 major domains: risk of bias (4 items) and applicability concerns (3 items). The items considered in both domains are: patient selection, index test and reference standard. The flow and timing item is only evaluated in terms of risk of bias. According to the responses to the landmark questions, each item can be scored “low”, “high”, or “uncertain” based on the risk of bias or on the concerns about the degree of matching with the review question. QUADAS-2 assessment was performed by two independent reviewers (MMo and MA). When possible, disagreements were resolved by consensus or otherwise adjudicated by a third member of the review team (SF).

### Statistical analysis

Statistical analyses were performed using Comprehensive Meta-analysis (Version 2, Biostat, Englewood NJ, 2005), Review Manager software (Version 5.4.1, The Cochrane Collaboration, Copenhagen, Denmark), R Statistical software (R Core Team 2021) and MetaDTA (Version 2.0) [23].

In each group, the response rate to ICS therapy in cases and controls was calculated as (number of responders)/(total number of subjects). The attributable proportion was defined as (response rate in cases – response rate in controls)/(response rate in cases) [24]. Differences in the response rates between cases and controls were expressed as diagnostic Odds Ratio (OR) with pertinent 95% confidence intervals (95%CI). Differences in FeNO values among responders and non-responders were expressed in parts per billion (ppb) as mean difference (MD) and 95%CI. The overall effect was tested using Z-scores, with a *p* < .05 being considered statistically significant. Since significant heterogeneity is to be expected in diagnostic accuracy studies [25], the random effects method was used whenever applicable to be as conservative as possible.

The bivariate random-effects binomial model was employed to obtain the summary point for sensitivity, specificity, positive (PLR) and negative likelihood ratio (NLR) with 95%CI [25]. The hierarchical summary receiver operating characteristic (HSROC) parameters and the area under the curve (AUC) were estimated using the bivariate model parameters and the equivalence equations of Harbord *et al* [26]. The Fagan’s nomogram was used to infer post-test probability from likelihood ratios and evaluate the clinical applicability of FeNO in a realistic scenario [27].

### Statistical heterogeneity

Statistical heterogeneity between studies was evaluated by using chi-square Cochran’s Q test and I^2^ index (non-threshold effect). The latter measures the inconsistency across study results and describes the proportion of total variation in study estimates, which is due to heterogeneity rather than sampling error. In detail, I^2^ values of 0% indicate no heterogeneity, 25% low, 25–50% moderate, and > 50% high heterogeneity [28]. The Spearman correlation coefficient between sensitivity and false positive rate was used to test for the presence of the threshold effect as a source of heterogeneity. A *p* > .05 indicated no threshold effect [29].

### Risk of bias assessment

Based on the analysis of the funnel plot of the effect size *vs.* the inverse of the square root of the effective sample size, the presence of publication bias was tested with the Deeks funnel plot asymmetry test [30].

Possible small-study effect was also addressed by performing a visual inspection of the funnel plots of the logarithmic effect size *vs.* precision (1/standard error of the logarithmic effect size) or of the mean difference *vs.* precision (1/standard error of the mean difference). The Egger’s and the Begg and Mazumdar tests were used to test for funnel plot asymmetry over and above any subjective evaluation. [31]. Finally, the Duval and Tweedie’s trim-and-fill analysis was performed to evaluate an adjusted effect size after trimming and imputing studies [32]. A *p* < .10 was considered statistically significant when assessing the risk of bias [33].

### Sensitivity analyses

Potential sources of heterogeneity were investigated by repeating the analyses after excluding retrospective studies. Because of the potential influence of smoking on FeNO values [34], we performed a further sensitivity analysis excluding the studies enrolling smokers. Moreover, we planned to separately analyse articles specifically using the recommended higher than 8-week limit for chronic cough definition [2]. Finally, we planned to repeat analyses after excluding any study with a high risk of bias and/or applicability concerns for patient selection according to QUADAS-2.

### Subgroup analyses

The ATS strongly recommend that FeNO values lower than 25 ppb should be considered irrelevant for clinical applications, implying non-eosinophilic or no airway inflammation [35]. Thus, since included studies used different thresholds, we separately analysed data based on the presence/absence of a FeNO cut-off greater than 25 ppb.

### Meta-regression analyses

We also evaluated the impact of demographic variables (mean age, male gender) and clinical characteristics of the study population related to body composition [body mass index (BMI)], smoking habit, atopic status, concomitant respiratory diseases [asthma, recent respiratory tract infection (RTI)], pulmonary function [forced expiratory volume in 1 s (FEV_1_), forced expiratory volume in 1 s/forced vital capacity (FEV_1_/FVC)], use of medications [angiotensin converting enzyme-inhibitors (ACE-I)], length of follow-up after ICS initiation, and cough duration on differences in the prevalence of steroid response between patients with high and low FeNO. Thus, we performed meta-regression analyses after implementing a regression model with the rate of response as dependent variable (y), and the above reported covariates as independent variables (x). Comprehensive Meta-analysis (Version 2, Biostat, Englewood NJ, 2005) was used for meta-regressions.

## Results

After excluding duplicate results, the search identified 2,237 articles. Of them, we excluded 1,993 because off topic after evaluating the title and the abstract, and 231 because were reviews/book chapters/editorials. Other 4 studies were excluded after the evaluation of the full length paper.

In the final analysis, we included 9 articles [17–20,36–40], in which a total of 740 chronic cough patients were enrolled (Supplemental Figure 1).

### Study characteristics

Table 1 reports the baseline demographic and clinical data of patients with chronic cough treated with ICS from the included studies. Characteristics of the study design and steroid treatment are reported in Table 2.

**Table 1.** Baseline demographic and clinical data of patients with chronic cough treated with inhaled corticosteroids (ICS) in included studies.

Study	High FeNO (*n*)	Low FeNO (*n*)	Males (%)	Age (years)	BMI (kg/m^2^)	Smoking (%)	Recent RTI (%)	ACE-I (%)	Asthma (%)	Atopic status (%)	ICS naïve (%)	FEV_1_ (% predicted)	FEV_1_/FVC
Hahn 2007	41	23	40.6	46.8	28.9	15.6^b^	–	0	48.4^c^	–	–	95.6	–
Hsu 2013	43	1	47.7	49.1	–	0	–	–	0	–	–	90.2	81.4
Koskela 2013^a^	15	24	25.5	55.6	27.4	0	0	–	20.9^d^	32.6	–	93.7	–
Lamon 2019	41	22	38.1	59.2	25.7	30.1^b^	–	–	9.5^e^	12.7	–	95.7	72.8
Price 2018^a^	31	65	47.4	50.0	27.6	9.6	0	0	54.8	–	–	90.8	78.0
Prieto 2009	19	24	43.4	48.0	–	0	0	0	9.3^c^	100	100	113.2	81.9
Shebl 2020	41	29	41.4	46.7	30.1	0	–	–	–	–	–	86.2	88.9
Watanabe 2016	21	56	44.1	47.8	–	51.9	13.0	–	50.6	5.2	55.8	–	–
Yi 2016	139	105	53.7	39.8	–	0	0	–	28.3	54.7^f^	–	96.0	80.4

Pop: population; BMI: body mass index; RTI: respiratory tract infection; ACE-I: angiotensin converting enzyme-inhibitors; FEV_1_: forced expiratory volume in 1 s; FVC: forced vital capacity. Continuous data are expressed as mean values, unless otherwise indicated.

^a^Baseline demographic and clinical data refer to an original population of 43 patients for Koskela 2013 and 114 patients for Price 2018.

^b^Any smoking history (previous or current).

^c^Asthma defined by methacholine challenge tests.

^d^Asthma defined by questionnaires.

^e^Asthma defined by positive anamnesis.

^f^Only 95 patients with available atopy data.

**Table 2.** Characteristics related to study design and inhaled corticosteroids (ICS) treatment in included studies.

Study	Study design	Definition of chronic cough	Cough duration	ICS type/dose	Follow-up	Definition of ICS response
Hahn 2007	Retrospective	≥8 weeks	40.3 months	Fluticasone propionate 434 μg/day	5.3 months	Physician-documented significant improvement in cough, no further diagnostic study ordered for assessment of cough, and no alteration in ICS dose
Hsu 2013	Retrospective	≥8 weeks	15.3 months	Fluticasone propionate 250 μg twice/day	≥2 weeks	Complete control of cough determined by physician
Koskela 2013	Prospective	≥8 weeks	8.5 years	Budesonide 400 μg twice/day	12 weeks	Improvement o*f* > 1.3 points in the Leicester Cough Questionnaire score
Lamon 2019	Retrospective	≥8 weeks	43.1 months	Unspecified ICS 1,020 μg/day	≥3 months	Self-reported reduction of cough frequency
Price 2018	Double-blind randomised placebo-controlled trial	≥6 weeks	–	Beclomethasone dipropionate 800 μg/day	4 weeks	Improvement o*f* ≥ 20 mm in the VAS cough score
Prieto 2009	Prospective	≥8 weeks	–	Fluticasone propionate 100 μg twice/day	4 weeks	Reduction o*f* > 50% in the mean daily cough symptom scores
Shebl 2020	Prospective	≥8 weeks	–	–	4 weeks	Complete cough disappearance according to a cough symptom score^a^
Watanabe 2016	Retrospective	≥3 weeks	15.4 months	–	3 months	Significant improvement in cough with ICS declared by the patient and confirmed by the physician
Yi 2016	Prospective	≥8 weeks	–	–	–	Complete control of cough determined by physician^b^

LCQ: Leicester Cough Questionnaire; VAS: Visual Analogue Scale. Continuous data are expressed as mean values, unless otherwise indicated.

^a^Information from another reference by the same authors [41].

^b^Information from another reference by the same authors [42].

The number of enrolled patients with chronic cough ranged between 39 and 244, with a mean-age ranging from 39.8 to 59.2 years, the mean BMI from 25.7 to 30.1 kg/m^2^, and the male gender prevalence from 38.1 to 53.7%. Asthma was documented in 0–54.8% of patients, an atopic status in 5.2–100%, a recent RTI in 0–13.0%, and a smoking history in 0–51.9%. Mean values of FEV_1_ ranged from 86.2 to 113.2% predicted, while the FEV_1_/FVC ratio was between 72.8 and 88.9. Data on the use of ACE-I and on the proportion of steroid naïve patients lacked in most included studies.

Four studies were retrospective [17–19,37], four were prospective [20,36,38,40], and one had a randomised double-blind placebo-controlled design [39]. With the only exception of 2 articles [37,39], all included studies complied with the definition of chronic cough as the presence of the symptom for at least 8 weeks [2]. Mean follow-up after starting ICS therapy ranged from a minimum of 2 weeks to 5.3 months.

Although the standardised procedures for FeNO assessment were followed in all the included studies, different cut-off values (from 16.3 ppb to 44.5 ppb) were used to discriminate high and low FeNO (Supplemental Table 1).

### Quality of the included studies

**Figure 1.**
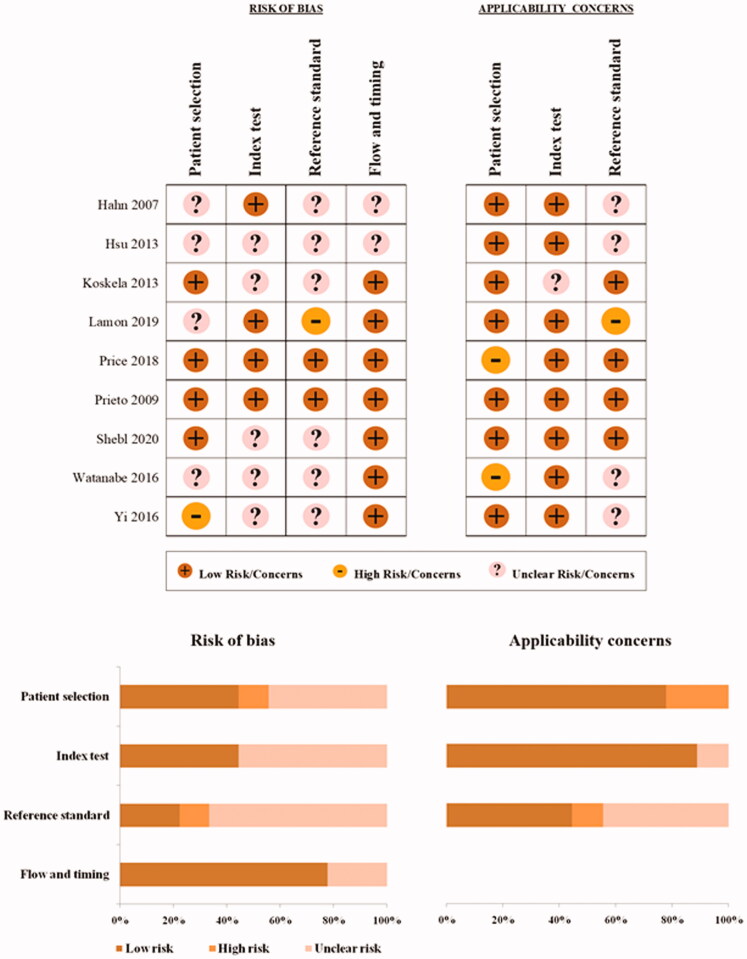
Quality of included studies according to Quality Assessment of Diagnostic Accuracy Studies 2 (QUADAS-2) criteria.

Figure 1 shows the QUADAS-2 report for each included study. Only one study presented a low risk of bias or low applicability concerns in all QUADAS-2 domains [20]. Four studies [17–19,37] with a retrospective design had an unclear risk of bias regarding patient selection. One prospective study [38] did not avoid a case-control design and was considered at high risk of bias for this item. Applicability concerns for patient selection were raised for two studies [37,39], which included patients with non-specific respiratory symptoms and/or used a lower than 8-week limit for chronic cough definition. Considering that baseline FeNO was assessed before starting ICS therapy, the index test results were interpreted without knowledge of the results of the reference standard in all included studies. With only one exception [36], all studies clearly described the devices and procedures for FeNO assessment. However, the FeNO cut-off value was not pre-specified in five articles [19,36–38,40], which were considered at unclear risk of bias for the index test item. Two studies [20,39] used validated scores as reference standard in blinded conditions, thus were judged at low risk of bias and with no applicability concerns for this item. Two further studies using validated scores [36,40] and four studies relying on the judgement of physicians to define ICS response [17,19,37,38] did not clearly report if the reference standard results were interpreted without knowledge of the results of the index test. Thus, we scored an unclear risk of bias for this item. Furthermore, unclear applicability concerns were raised for studies [17,19,37,38] not using a validated and repeatable tool to assess ICS response. Only one study [18] based the effectiveness of steroids on a patient declaration of cough frequency reduction, thus being scored at high risk of bias with high concerns in terms of applicability. Exclusion rates among retrospective studies were high in two studies [17,19], which were scored at unclear risk of bias in terms of flow and timing.

### Performance of FeNO in predicting ICS response

**Figure 2.**
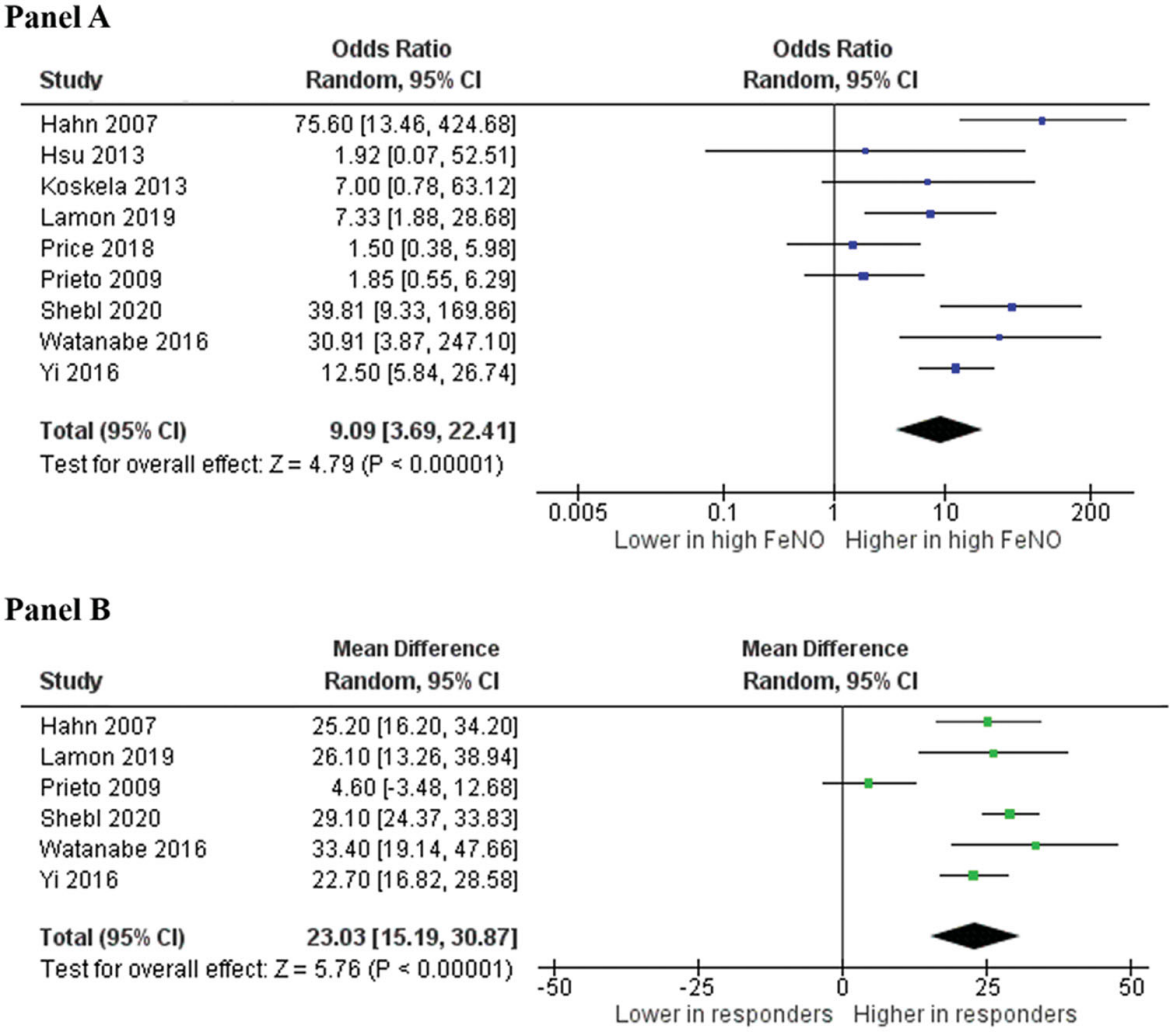
Forest plots of cough response to inhaled corticosteroids in patients with high and low fractional exhaled nitric oxide (A) and of the mean difference in fractional exhaled nitric oxide between responders and non-responders (B). FeNO: fractional exhaled nitric oxide; 95%CI: 95% confidence interval. Squares represent the Odds Ratio of steroid response in chronic cough patients with a high and low FeNO in each study (A) or the Mean Difference in FeNO between responders and non-responders to corticosteroids. Lines are the 95% confidence intervals. The black diamond represents the cumulative Odds Ratio (A) or the cumulative Mean Difference (B) for analysed studies.

The analysis of the nine studies [17–20,36–40] showed that the response rate to ICS was 87.4% (95%CI: 83.8–91.0) in 317 chronic-cough patients with a high FeNO, and 46.3% (95%CI: 41.6–51.0) in 423 controls, with an attributable proportion of 47.0% and a corresponding OR of 9.1 (95%CI: 3.7–22.4, *p* < .001, Figure 2(A)). The heterogeneity among the studies was significant (I^2^: 69.0%, *p* = .001) and was not reduced by the exclusion of one study at a time.

**Figure 3.**
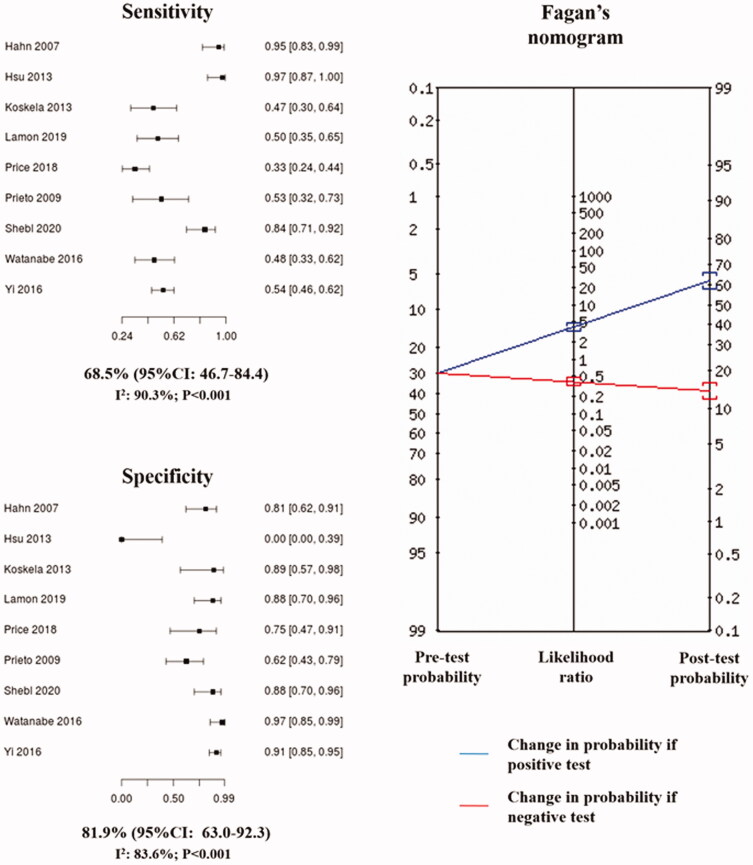
Pooled estimates of sensitivity and specificity for the association between high fractional exhaled nitric oxide (FeNO) and response to inhaled corticosteroids in chronic cough, with Fagan’s nomogram from pooled likelihood ratios. 95%CI: 95% confidence interval. Squares represent the sensitivity or specificity for the association between high FeNO and response to inhaled corticosteroids. Lines are the 95% confidence intervals.

**Figure 4.**
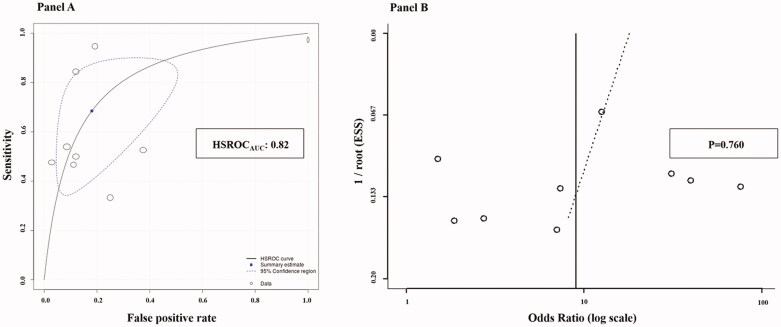
Hierarchical Summary Receiver Operating Characteristic (HSROC) curve (A) and Deeks test for funnel plot asymmetry (B). HSROC_AUC_: area under the HSROC curve; ESS: effective sample size.

The pooled analysis of diagnostic indexes resulted in a sensitivity of 68.5% (95%CI: 46.7–84.4) and a specificity of 81.9% (95%CI: 63.0–92.3), with a PLR of 3.79 (95%CI: 1.24–7.47) and a NLR of 0.38 (95%CI: 0.22–0.66). Based on pooled likelihood ratios and a pre-test probability set at 30% by default, Fagan’s nomogram revealed that the post-test probability increased to 62% if the patient had a high FeNO and decreased to 14% if the test was negative (Figure 3). The analysis of the HSROC curve revealed a pooled AUC of 0.82 (95%CI: 0.64–0.90) (Figure 4(A)). No threshold effect was detected by diagnostic threshold analysis, confirmed by a Spearman correlation coefficient between sensitivity and false positive rate of 0.498 (*p* = .173).

### Publication bias

Since publication bias has been reported to affect results of meta-analyses, this potential bias was assessed by using funnel plots analysis. The Deeks funnel plot asymmetry test suggested the absence of publication bias, confirmed by a *p*-value of 0.760 (Figure 4(B)). Accordingly, the funnel plot of the effect size *vs.* precision for studies evaluating the difference in steroid response between chronic-cough patients with high and low FeNO was rather symmetrical, excluding the presence of publication bias and of small-study effect (Egger’s *p* = .995). The absence of significant publication bias was further confirmed by the Begg and Mazumdar test as well as by the Duval and Tweedie’s trim and fill analysis (Supplemental Figure 2).

### Sensitivity analyses

Study design can potentially influence the observed results. Therefore, we repeated the analysis after excluding the four retrospective studies [17–19,37], confirming, however, the results (OR: 6.3; 95%CI: 1.9–20.8, *p* < .001; I^2^: 76.5%, *p* = .002). Similarly, when specifically considering the studies on non-smokers [19,20,36,38,40], we obtained an OR of 7.8 (95%CI: 2.4–25.0, *p* < .001; I^2^: 66.8%, *p* = .017) for the association between high FeNO and steroid response. A diagnostic OR of 10.5 (95%CI: 4.0–27.5, *p* < .001; I^2^: 66.0%, *p* = .007) was documented after excluding 2 studies [37–39] using a lower than 8-week limit for chronic cough definition. Overall, after excluding 3 studies [37–39] with a high risk of bias and/or applicability concerns for patient selection, an OR of 10.0 (95%CI: 2.7–36.4, *p* < .001; I^2^: 71.1%, *p* = .004) was confirmed.

Results substantially comparable to those of the overall analysis with no threshold effect were obtained when estimating the pooled diagnostic indexes for these sensitivity analyses (Table 3).

**Table 3.** Pooled diagnostic indexes for the association between high fractional exhaled nitric oxide (FeNO) and response to inhaled corticosteroids in sensitivity and subgroup analyses.

	SENSITIVITY ANALYSES				SUBGROUP ANALYSES	
	Exclusion of retrospective studies	Exclusion of data on smokers	Exclusion of studies not using the recommende*d* ≥ 8-week limit for chronic cough definition	Exclusion of studies with high risk of bias and/or applicability concerns in patient selection	FeNO cut-of*f* ≤ 25 ppb	FeNO cut-of*f* > 25 ppb
Sensitivity (%)	54.3 (95%CI: 37.3–70.5)	64.2 (95%CI: 58.2–69.9)	76.0 (95%CI: 52.2–90.1)	79.2 (95%CI: 53.0–92.8)	49.6 (95%CI: 39.0–60.2)	77.4 (95%CI: 46.5–93.1)
Specificity (%)	83.6 (95%CI: 70.7–91.5)	83.4 (95%CI: 77.0–88.7)	78.1 (95%CI: 53.8–91.6)	73.9 (95%CI: 43.6–91.2)	79.7 (95%CI: 60.2–91.1)	81.3 (95%CI: 50.2–94.9)
PLR	3.31 (95%CI: 1.51–7.28)	2.55 (95%CI: 0.83–7.80)	3.47 (95%CI: 1.65–7.30)	3.04 (95%CI: 1.31–7.05)	2.44 (95%CI: 1.12–5.33)	4.14 (95%CI: 1.50–11.41)
NLR	0.55 (95%CI: 0.35–0.84)	0.36 (95%CI: 0.20–0.67)	0.31 (95%CI: 0.16–0.61)	0.28 (95%CI: 0.12–0.64)	0.63 (95%CI: 0.48–0.84)	0.28 (95%CI: 0.11–0.69)
HSROC_AUC_	0.77 (95%CI: 0.53–0.91)	0.78 (95%CI: 0.54–0.91)	0.83 (95%CI: 0.55–0.90)	0.82 (95%CI: 0.64–0.89)	0.52 (95%CI: 0.47–0.63)	0.83 (95%CI: 0.62–0.94)

PLR: positive likelihood ratio; NLR: negative likelihood ratio; SROC_AUC_: area under the summary receiver operating characteristic curve; Q* index: intercept of the summary receiver operating characteristic curve; HSROC_AUC_: area under the hierarchical summary receiver operating characteristic curve.

### Subgroup analyses

The subgroup analysis of the three studies [18,20,36] using a lower FeNO threshold (≤ 25 ppb) suggested a lower performance in predicting ICS response (OR: 3.9; 95%CI: 1.5–10.3, *p* = .010; I^2^: 20.6%, *p* = .284), confirmed by the estimate of pooled diagnostic indexes and an AUC of 0.52 (95%CI: 0.47–0.63).

In contrast, when separately analysing studies [17,19,37–40] with a FeNO cut-off greater than 25 ppb, a better performance was documented (OR: 13.5; 95%CI: 4.1–44.2, *p* < .001; I^2^: 71.6%, *p* = .003) with an AUC of 0.83 (95%CI: 0.62–0.94) (Table 3). To assess the clinical utility of the recommended FeNO threshold [35], the Fagan’s nomogram was used, revealing a post-test probability of 64% if the patient had a high FeNO and 11% if the test was negative.

### Meta-regression analyses

As shown in Supplemental Table 3, no demographic or clinical variable related to body composition, smoking habit, atopic status, concomitant respiratory diseases, pulmonary function, length of follow-up and cough duration affected the difference in steroid response rate between chronic cough patients with high and low FeNO values.

### FeNO values among responders and non-responders

In line with the above results, in six studies [17,18,20,37,38,40] we found that 321 chronic cough patients responding to ICS therapy showed significantly higher FeNO values as compared to 240 non-responders (MD: 23.0 ppb; 95%CI: 15.2–30.9, *p* < .001; I^2^=83.0%; *p* < .001, Figure 2(B)). Of interest, when excluding the study by Prieto et al. [20], an even higher difference between responders and non-responders was observed (MD: 26.7 ppb; 95%CI: 23.5–29.4, *p* < .001) without heterogeneity (I^2^=0%; *p* = .440).

The analysis of the funnel plot for studies evaluating FeNO levels among responders and non-responders to ICS suggested the absence of publication bias for this additional analysis, also confirmed by the Egger’s test (*p* = .917).

## Discussion

The results of our meta-analysis suggest that high FeNO values before starting ICS therapy may be associated with higher response rates in chronic cough patients, with an acceptable performance in predicting steroid responsiveness. These findings are supported by sensitivity and meta-regression analyses, showing no impact of several clinical and demographic variables on the observed results, including those related to cough aetiology and duration. Furthermore, this meta-analysis offers interesting insights into the identification of the optimal FeNO cut-off for predicting response to steroids. In particular, in line with the ATS recommendations [35], we documented a better performance of FeNO values higher than 25 ppb, with a difference of 23 ppb between responders and non-responders.

In recent years, FeNO has become a useful and non-invasive method to identify chronic inflammation of airway diseases [43], thus being successfully used to monitor adherence to steroid treatment in asthmatic patients [44]. Considering that steroids are able to reduce eosinophilic inflammation documented in most asthma phenotypes, FeNO is the perfect biomarker for monitoring steroid response in this clinical setting [44]. In patients with mild-to-moderate asthma, a recent meta-analysis demonstrated that a gradual ICS reduction when FeNO is below 50 ppb does not increase exacerbations [45]. The effectiveness of FeNO for asthma management was also confirmed when specifically evaluating paediatric populations [46]. Moreover, FeNO has been considered for asthma diagnostics, with values greater than 40 ppb showing the best performance (OR: 9.8) [47]. More recently, the need for a safe and simple method driving the decisional process of steroid prescription in patients with non-specific respiratory symptoms has led to a growing focus on this biomarker [7]. Thus, randomised studies with a more robust design have been carried out to better address this issue [39].

Considering that cough-variant asthma, eosinophilic bronchitis and atopic cough are characterised by a T helper 2-mediated airway inflammation, they more often tend to respond to ICS treatment [44]. However, ICS responsiveness can depend upon several factors, including direct and indirect smoking exposure [48] and cough duration [49]. In keeping with this, a number of studies failed in demonstrating a correlation between airway hyperresponsiveness and ICS response in patients with subacute or chronic cough [48,50]. A recent meta-analysis of randomised clinical trials reported only a modest efficacy of ICS over placebo on chronic cough, thus concluding that a more rigorous patient selection is needed to identify those who may certainly respond to ICS [51]. Overall, the diagnostic uncertainty often leads to inappropriate ICS prescription, particularly in subjects with undiagnosed non-specific respiratory symptoms [52], and in those with recent RTI and otherwise self-limiting cough [53].

The rationale for using FeNO in the decisional process of ICS prescription is complex and only partially elucidated [44]. In brief, eosinophilic airway inflammation derives from the activation of mast cells and antigen-specific T helper 2 cells, with the concomitant production of cytokines, such as interleukin (IL)-4, IL-5 and IL-13 [10]. Their release regulates the expression of the inducible isoform of NO synthase (iNOS), thus determining an increased NO production in an allergic environment [11]. This implies that FeNO assessment may be able to accurately predict eosinophilic airway inflammation - a peripheral trigger of cough reflex with a good ICS response - in a rapid, non-demanding and cost-effective manner [54]. In a previous cost-effectiveness analysis, Sabatelli et al found that combining FeNO monitoring with standard asthma care saved €62.53 ($74.40) per patient-year in Spain, with potential annual savings of €129 million ($153 million) [55]. A similar conclusion was obtained for the asthma management in paediatric population [56].

Overall, our findings partly respond to the urgent need of rapid and reliable methods to identify chronic cough patients who could benefit from steroid therapy. In a previous systematic review, Song et al concluded that there is no strong evidence to support the use of FeNO testing for predicting ICS responsiveness in chronic cough [7]. However, this interesting study that first appeared online in 2016, although rigorously conducted according to PRISMA guidelines, identified articles published in peer-reviewed journals up to February 2015, thus including ≈50% of currently available studies. Thus, given the low number of studies and the considerable heterogeneity among the study designs and outcome measurements, the Authors conveniently decided not to carry out any meta-analytical evaluation. Considering the higher number of available articles in 2021, an attempt was made to perform a meta-analysis, although aware of the relevant limitations due to the high heterogeneity and the impossibility of drawing definitive conclusions. In fact, statistical heterogeneity was found to be generally significant as a result of our analyses, potentially depending on a different study design and different inclusion and exclusion criteria in considered studies. For example, a diagnostic accuracy study should ideally enrol a random or consecutive sample of eligible patients with the suspected condition to avoid the case-control design and, therefore, a potential bias. Thus, the inclusion of studies enrolling patients with the target condition and a control group without it may overestimate diagnostic accuracy [57]. To improve the critical evaluation of published studies, the QUADAS-2 tool was specifically designed, thus ensuring greater transparency in the classification of bias and applicability for diagnostic accuracy meta-analyses [22]. In our meta-analysis, based on this quality assessment of included studies, we planned a number of additional analyses to investigate potential sources of such heterogeneity, including a sensitivity analysis without the studies at high risk of bias for patient selection. Of interest, our results were substantially confirmed in each of these additional analyses. Similarly, meta-regression analyses were also planned to evaluate the influence of a number of clinical and demographic variables on the observed results. For example, the length of follow-up after ICS initiation and the mean duration of chronic cough were quite heterogeneous in different studies and this may have significantly affected our findings [46]. The observation that such demographic and clinical covariates – even those related to cough aetiology (*e.g.* asthma, atopy, smoking) and duration – had no impact on our results confirms the potential usefulness of FeNO in predicting ICS response.

Overall, in our meta-analysis, we reported a significantly higher response rate in high FeNO patients as compared to those with a low FeNO (87.4% *vs*. 46.3%), corresponding to a diagnostic OR of 9.1. Of interest, in line with the ATS recommendation [35], the performance of this biomarker was even higher (OR: 13.5) when specifically considering studies with a FeNO cut-off greater than 25 ppb. Unfortunately, because of the relatively low number of included studies (*n* = 9), no further stratification according to different cut-off values could be performed. In our opinion, the fact that different devices for FeNO assessment were used in different studies had no effect on our results, since all included studies substantially followed ERS/ATS standardised procedures [12–14], although with different thresholds. Interestingly, the threshold effect was not the source of heterogeneity of our meta-analysis. Thus, considering that likelihood ratios do not change with the pre-test probability [58], the estimated post-test probabilities in a more realistic scenario suggested that the test could be considered suitable for routine practice. The usefulness of FeNO in the decisional process of ICS prescription has been recently reported also in chronic respiratory diseases [59,60], thus suggesting that this biomarker could be considered in the near future for a routine use in a wide range of clinical and healthcare settings (*i.e.* community, hospital, rehabilitation) [61,62].

As reported above, our meta-analysis has relevant limitations, mainly due to the considerable heterogeneity among the study designs and outcome measurements, with no objective information regarding the baseline severity of cough or magnitude of improvement of cough. Since a meta-analysis is performed on aggregate data and some missing information is present in each study, our sensitivity, subgroup and meta-regression analyses were able to refine results, allowing for the adjustment for some – but not all – potential confounders. Moreover, the presence of publication bias was consistently excluded with different methods. Overall, despite the additional analyses substantially confirming our findings, caution is required when interpreting the results of this meta-analysis. Over and above the aforementioned limitations, the relatively low number of included studies with a small sample should be considered as a major weakness of our pooled analyses, confirmed by the large confidence intervals reflecting the uncertainty in the estimation of the diagnostic test accuracy.

In conclusion, our results suggest that a high FeNO before starting ICS therapy may help identify chronic cough patients responding to treatment, with a better performance for higher cut-off values. This further supports the need for large clinical trials with a robust design aimed at defining more refined prediction strategies before ICS initiation, in order to guide decisions and reduce the risk of adverse events and cost-ineffective strategies. In this regard, FeNO may have the potential to be used as an additional tool to identify combined prediction strategies allowing for more individualised approaches.

## Supplementary Material

Supplemental Material

## Data Availability

The data that support the findings of this study are available from the corresponding author [M.M.] upon reasonable request.
