# Supplementary material for: Performance of fractional exhaled nitric oxide in predicting response to inhaled corticosteroids in chronic cough: a meta-analysis
Source: Ann Med. 2021 Sep 16;53(1):1659–72. doi: 10.1080/07853890.2021.1979242 (PMC8451665; doi:10.1080/07853890.2021.1979242)
Supplement: Supplemental Material [file IANN_A_1979242_SM0530.doc]

**Supplementary online material**

**Performance of fractional exhaled nitric oxide in predicting response**

**to inhaled corticosteroids in chronic cough: a meta-analysis.**

Pasquale Ambrosino, **MD PhD**^1^ Mariasofia Accardo, **MD**^1^

Marco Mosella, **MD**^1^ Antimo Papa, **MD**^1^ Salvatore Fuschillo, **MD**^1^

Giorgio Alfredo Spedicato, **PhD FCAS FSA CSPA CStat**^2^

Andrea Motta, **PhD**^3^ Mauro Maniscalco, **MD PhD**^1^

^1^Istituti Clinici Scientifici Maugeri IRCCS, Pavia, Italy;

^2^Department of Data Analytics and Actuarial Science, Unipol Group, Bologna, Italy

^3^Institute of Biomolecular Chemistry, National Research Council, ICB-CNR, Pozzuoli, Naples, Italy.

**Table of contents**

| **Supplemental Table 1** | **Devices and methods for measurement of fractional exhaled nitric oxide (FeNO) in included studies.** |
| --- | --- |
| **Supplemental Table 2** | **Meta-regression analyses. Impact of clinical and demographic characteristics on the difference in steroid response rate between chronic cough patients with high and low fractional exhaled nitric oxide (FeNO).** |
| **Supplemental Table 3** | **PubMed search (June 8, 2021).** |
| **Supplemental Figure 1** | **Preferred Reporting Items for Systematic Reviews and Meta-Analyses (PRISMA) flow diagram.** |
| **Supplemental Figure 2** | **Funnel plot of the logarithmic effect size *vs.* precision (1/standard error of the effect size) for studies evaluating the difference in steroid response between chronic cough patients with high and low fractional exhaled nitric oxide.** |

**Supplemental Table 1.** **Devices and methods for measurement of fractional exhaled nitric oxide (FeNO) in included studies.**

| **Study** | **Analyzer** | **Expiratory flow rate**  **(ml/s)** | **Commercial equipment** | **Cut-off value**  **(ppb)** |
| --- | --- | --- | --- | --- |
| Hahn 2007 | Chemiluminescence | 50 | - Sievers NOA-280i®, GE Analytical Instruments (Boulder, USA) | 35 |
| Hsu 2013 | Chemiluminescence | 50 | - Sievers NOA-280i®, GE Analytical Instruments (Boulder, USA) | 30 |
| Koskela 2013 | - | - | - - | 16.3 |
| Lamon 2019 | Electrochemical | 50 | - NIOX MINO Airway Inflammation Monitor®, Aerocrine AB (Solna,Sweden) | 25 |
| Price 2018 | Electrochemical | 50 | - NIOX VERO®, Circassia (Solna, Sweden) | 40 |
| Prieto 2009 | Chemiluminescence | 45 | - Sievers NOA-280i®, GE Analytical Instruments (Boulder, USA) | 20 |
| Shebl 2020 | Electrochemical | 50 | - NIOX MINO Airway Inflammation Monitor®, Aerocrine AB (Solna,Sweden) | 34.5 |
| Watanabe 2016 | Electrochemical | 50 | - NIOX MINO Airway Inflammation Monitor®, Aerocrine AB (Solna,Sweden) | 44.5 |
| Yi 2016 | Electrochemical | 50 | - NIOX MINO Airway Inflammation Monitor®, Aerocrine AB (Solna,Sweden) | 31.5 |

**Supplemental Table 2. Meta-regression analyses. Impact of clinical and demographic characteristics on the difference in steroid response rate between chronic cough patients with high and low fractional exhaled nitric oxide (FeNO).**

| **Variable** | **Score** |
| --- | --- |
| Male gender | Z-value: -0.309, P=0.757 |
| Age | Z-value: -0.698, P=0.485 |
| BMI | Z-value: 1.253, P=0.210 |
| Smoking | Z-value: 0.819, P=0.413 |
| Recent RTI | Z-value: 1.266, P=0.205 |
| ACE-I | N/A |
| Asthma | Z-value: 0.998, P=0.318 |
| Atopic status | Z-value: -1.765, P=0.077 |
| ICS naive | N/A |
| FEV_1_ | Z-value: -1.134, P=0.257 |
| FEV_1_/FVC | Z-value: 1.007, P=0.314 |
| Follow-up | Z-value: 1.620, P=0.105 |
| Cough duration | Z-value: -0.358, P=0.720 |

**BMI: body mass index; RTI: respiratory tract infection; ACE-I: angiotensin converting enzyme-inhibitors; FEV_1_: forced expiratory volume in 1 second; FVC: forced vital capacity; N/A: not evaluated because of the low number of studies reporting this covariate.**

**Supplemental Table 3. PubMed search (June 8, 2021).**

| **Search terms** | **Number of results** |
| --- | --- |
| (cough) | 65,734 |
| (cough) AND (nitric oxide) | 438 |
| (cough) AND (nitric oxide) AND (steroid) | 4,180 |
| (cough) AND (nitric oxide) AND (steroid OR steroids) | 43 |
| (cough) AND (nitric oxide) AND (steroid OR steroids OR corticosteroid) | 94 |
| (cough) AND (nitric oxide) AND (steroid OR steroids OR corticosteroid OR corticosteroids) | 94 |

**Supplemental Figure 1. Preferred Reporting Items for Systematic Reviews and Meta-Analyses (PRISMA) flow diagram.**

**
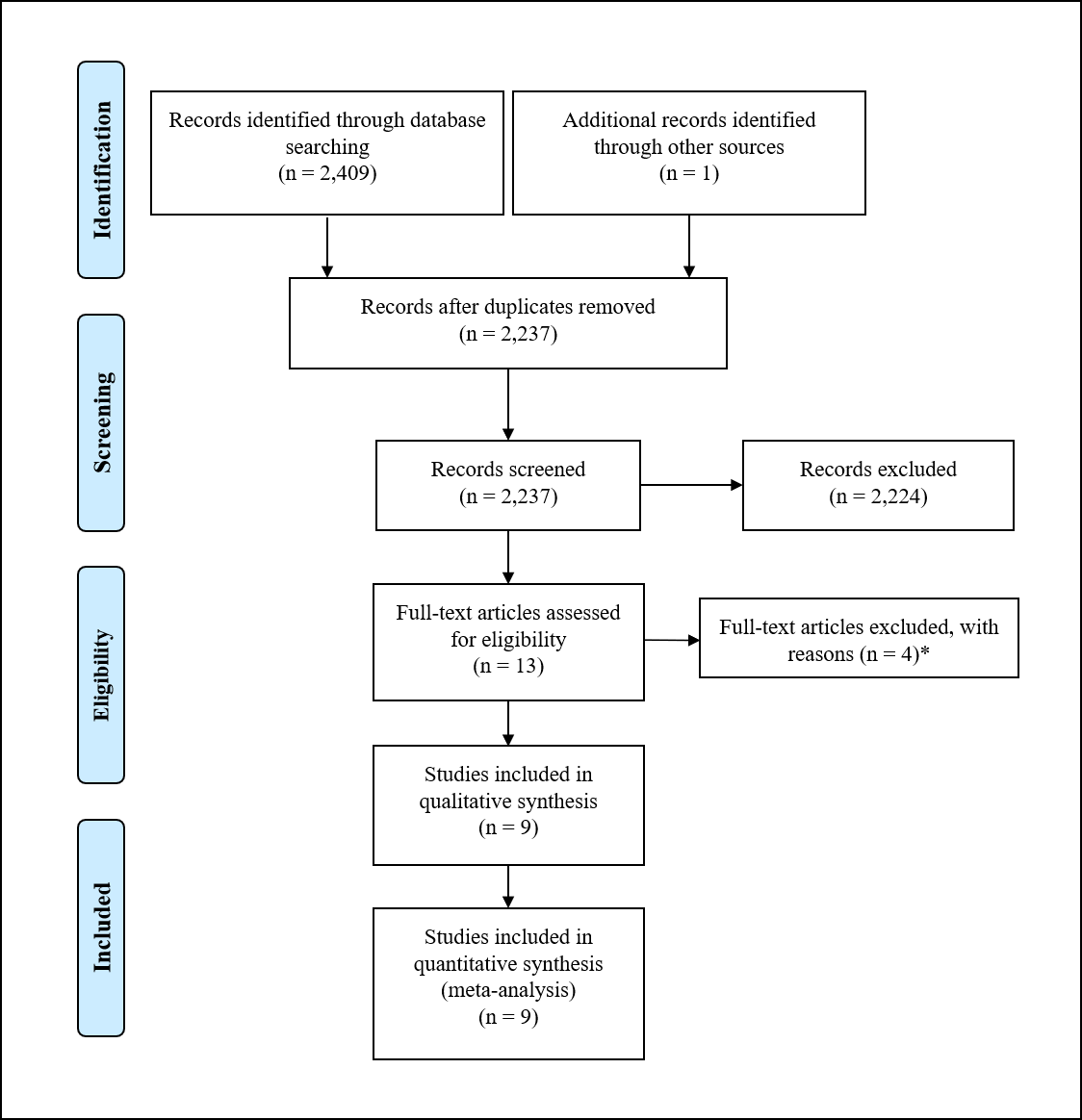
**

***3 studies with no data of interest, 1 study evaluating response to treatment according to Asthma Control Questionnaire 7 (ACQ7) score.**

**Supplemental Figure 2. Funnel plot of the logarithmic effect size *vs.* precision (1/standard error of the effect size) for studies evaluating the difference in steroid response between chronic cough patients with high and low fractional exhaled nitric oxide.**

**
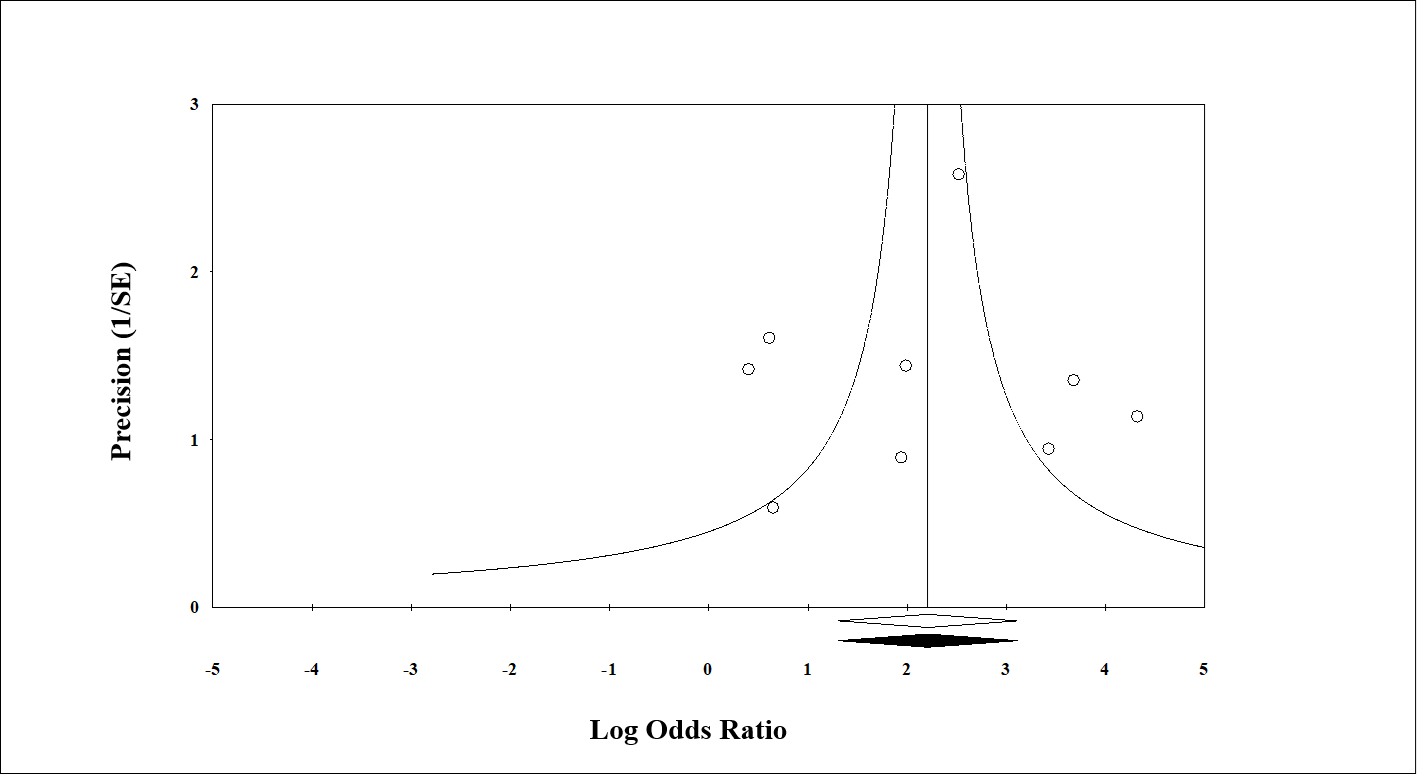
**

**SE: standard error. Observed studies and effect size are represented by empty circles and empty square. Imputed studies and adjusted effect size are represented by black circles and black square.**

| **Egger’s regressin intercept** | t-value | 0.00613 |  |
| --- | --- | --- | --- |
|  | df | 7.00000 |  |
|  | P-value | **0.99528** |  |
| **Begg and Mazumdar test** | **Kendall’s S statistic (P-Q)** | 4.00000 |  |
|  | **Kendall’s tau without continuity correction** |  |  |
|  | tau | 0.11111 |  |
|  | Z-value for tau | 0.41703 |  |
|  | P-value | **0.67666** |  |
|  | **Kendall’s tau with continuity correction** |  |  |
|  | tau | 0.08333 |  |
|  | Z-value for tau | 0.31277 |  |
|  | P-value | **0.75445** |  |

| **Duvall and Tweedie’s trim and fill** | **Studies**  **trimmed** | **Point**  **estimate** | **Lower**  **limit** | **Upper**  **limit** | **Q value** |
| --- | --- | --- | --- | --- | --- |
| Observed values | - | 9.08896 | 3.68951 | 22.39031 | 25.79348 |
| Adjusted values (on the left) | 0 | 9.08896 | 3.68951 | 22.39031 | 25.79348 |
| Adjusted values (on the right) | 0 | 9.08896 | 3.68951 | 22.39031 | 25.79348 |
